# Association between injury severity scores and clinical outcomes in patients with traumatic spinal injury in an aging Japanese society

**DOI:** 10.1097/MD.0000000000035369

**Published:** 2023-09-29

**Authors:** Manami Tsukuda, Gentaro Kumagai, Kanichiro Wada, Toru Asari, Yohshiro Nitobe, Eiichi Tsuda, Yasuyuki Ishibashi

**Affiliations:** a Department of Orthopedic Surgery, Hirosaki University Graduate School of Medicine, Hirosaki, Japan; b Department of Rehabilitation Medicine, Hirosaki University Graduate School of Medicine, Hirosaki, Japan.

**Keywords:** clinical outcomes, injury severity score, traumatic spinal injury

## Abstract

The Injury Severity Score (ISS) is widely used to evaluate patients with multiple injuries. This study investigated the association between ISS and clinical outcomes of patients with spinal cord injury (SCI) in an aging Japanese population. This retrospective cohort study investigated patients admitted to a Japanese university hospital. In the study, 89 patients with traumatic SCI were included. Traumatic SCI was categorized as monotrauma or polytrauma, and the ISS was used to evaluate trauma severity. Spearman’s correlation coefficient was used to estimate the correlation between ISS and the American Spinal Injury Association (ASIA) motor score, Barthel Index (activities of daily living assessment), and the European Quality of Life (QOL) scale (EQ5d) as an assessment of QOL at admission or the last follow-up with the adjustment for age, sex, and body mass index. Return to home and work were analyzed using the chi-squared test after the ISS was divided into three groups (<14, 14–19, and 20). The mean ISS was significantly higher for polytrauma than monotrauma. Significant negative correlations between the ISS and ASIA motor scores at the first visit (*P* < .001, *r* = −0.37) and the last follow-up (adjusted, *P* = .007, *r* = −0.30) were observed. The Barthel Index was also negatively correlated with ISS at the first visit (*P* = .04, *r* = −0.21) and at the last follow-up period (*P* < .001, *r* = −0.35). Moreover, ISS was significantly negatively correlated with EQ5d score at the last follow-up (*P* = .01, *r* = −0.28). The chi-squared test demonstrated that patients with an ISS of < 14 returned home (*P* = .03), while those with an ISS of < 19 returned to work (*P* = .02). ISS is associated with paralysis, activities of daily living, QOL, and lifestyle in patients with SCI and is an important initial injury assessment method.

## 1. Introduction

The characteristics of patients with spinal cord injury (SCI) in Japan changed between 1992 and 2018 as the population aged. The estimated incidence of traumatic SCI is 49 per million, of which cervical cord injuries were observed in 88.1% of the affected population. Additionally, spinal cord injuries without radiographic abnormalities (SCIWORA) impacted 62% of the affected population.^[1]^ Frankel grade D injuries accounted for most cases (46.3%). The most frequent cause of traumatic SCI was a fall from a level surface (38.6%).^[1]^

Polytrauma reportedly occurred in 55% of patients with traumatic SCI.^[2]^ The prevalence of thoracic lesions is higher in SCI patients with polytrauma than in those with SCI alone.^[2]^ Patients with polytrauma have more prolonged hospitalizations and regain activities of daily living at a slower pace.^[3]^ However, neurological recovery is associated with lesion completeness rather than the presence of polytrauma.^[2,3]^

The Injury Severity Score (ISS) is widely used to evaluate patients with multiple injuries.^[4,5]^ A study from Canada reported that the ISS was correlated with the total length of hospitalization and number of days of recumbency. In the same study, SCI was negatively correlated with the Functional Independence Measure score at discharge and 1 to 2 years after injury.^[6]^ In a previous report in the USA, in the acute phase, older patients had a higher ISS, more extended hospitalization periods, lower Glasgow Coma Scale scores, and significantly higher odds of being discharged to an institution rather than home as compared to young individuals.^[7]^ However, the association between the ISS and clinical outcomes in patients with SCI in an aging Japanese society remains unclear. Thus, the current study investigated the association between the ISS and clinical outcomes in patients with SCI at a single healthcare facility in Japan.

## 2. Materials and methods

### 2.1. Participants

**Figure 1.**
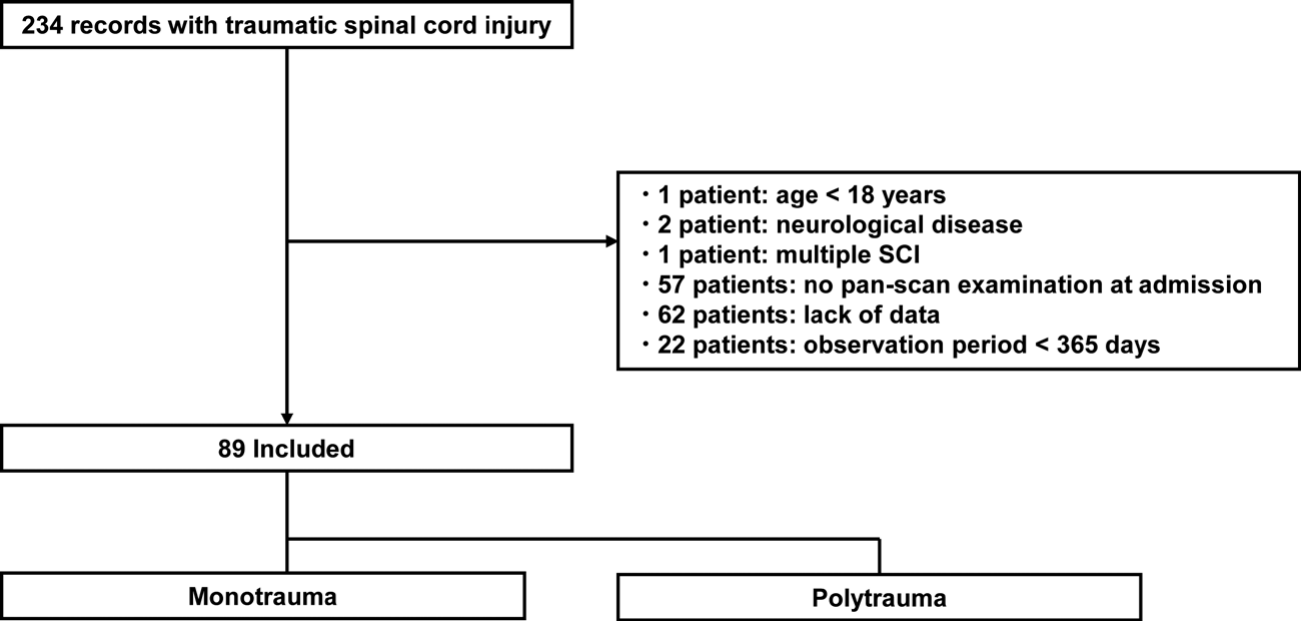
Flow diagram of the inclusion criteria. A total of 86 patients were included in this study, while 120 patients were excluded.

This retrospective cohort study adhered to the applicable STROBE guidelines^[8]^ and was conducted at a single tertiary emergency and critical care center in Japan. Data were collected from the medical records of patients with spinal trauma admitted to our orthopedic department between January 2011 and December 2021. Spinal trauma was diagnosed by surgeons in our department. The exclusion criteria were as follows: age < 18 years at the time of the injury; missing medical record data; comorbidities such as Parkinson’s disease and multiple SCI; no pan-scan examination at admission; and observation period < 365 days. This study included 89 patients who met the inclusion criteria (Fig. 1). This study received institutional review board approval from Hirosaki University School of Medicine and Hospital (Blind) (2018–1002), which waived the requirement for informed consent owing to data anonymity.

### 2.2. ISS and definition of polytrauma versus monotrauma

The anatomical regions with traumatic injuries were scored and categorized into 9 areas–head, face, neck, thorax, abdomen, spine, upper and lower limbs, and external–using the 2008 Abbreviated Injury Scale by referencing clinical records and computed tomography and magnetic resonance imaging scans at admission.^[9]^ Moreover, each patient was assessed for comprehensive severity using the ISS. Upon use of the Abbreviated Injury Scale to categorize the 6 body areas–head and neck, face, thorax, abdomen, extremities (including the pelvis), and external body–the ISS was calculated by adding the squares of the highest Abbreviated Injury Scale grades for each of the three most severely injured body regions. Traumatic SCI can be categorized as monotrauma or polytrauma. Polytrauma was defined using the Newcastle definition: Abbreviated Injury Scale code > 3 in at least 2 body regions.^[10]^ Monotrauma was defined as a spinal injury with an Abbreviated Injury Scale code ≤ 2 in other body regions.

### 2.3. Data collection

Demographic data included age, sex, body mass index, presence of polytrauma, medical history including ossification of the posterior longitudinal ligament and diffuse idiopathic skeletal hyperostosis, primary injury level, American Spinal Injury Association (ASIA) Impairment Scale,^[11]^ cause of injuries, SCIWORA, ISS, history of surgical treatment of the spine or polytrauma, transfer to a rehabilitation hospital, total length of hospitalization, total rehabilitation term, and observation period.

The primary outcomes were the correlation between ISS and ASIA motor score^[11]^ and Barthel Index^[12]^ at admission and the last follow-up period; European Quality of Life Score (EQ5d)^[13]^; living location (home, care facility, long-term medical care ward); and return to work by the last follow-up.

### 2.4. Statistical analysis

All statistical analyses were performed using SPSS version 29 (IBM, Chicago, IL). The data were not normally distributed. The Mann–Whitney *U* test was used to compare ordinally distributed data (such as age) between the monotrauma and polytrauma patients, and the median and standard deviation (SD) was calculated. The chi-squared test was used to compare the proportions of nominally distributed data (such as sex) between groups. Spearman’s correlation coefficient was used to estimate the correlation between ISS and neurological injury level (C2–C7, T1–T12, L1–L5) or outcomes such as ASIA motor score, Barthel Index at admission or the last follow-up, and EQ5d at the last follow-up after the adjustment for age, sex, and body mass index. Return to home and work were also analyzed using the chi-squared test after ISS was divided into three groups by score (<14, 14–19, and 20) according to a previous study.^[7]^ Statistical significance was set at *P* < .05. Values are presented as mean ± SD.

## 3. Results

### 3.1. Characteristics of SCI patients with monotrauma versus polytrauma

**Figure 2.**
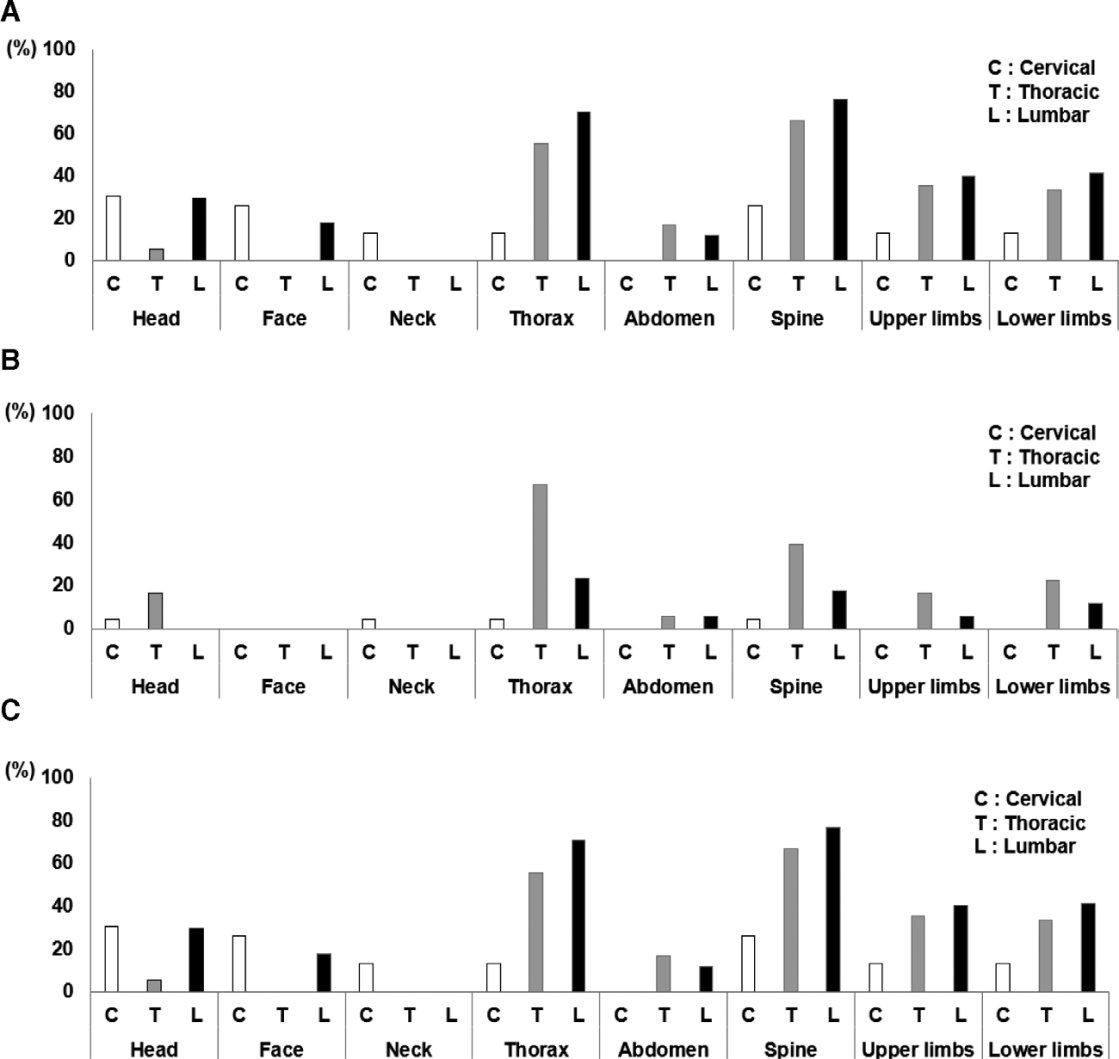
Association between spinal injury level and other body injuries. Head and face injuries are common with cervical and thoracic lesions. Neck injuries occurred with cervical lesions. Thoracic injuries were common with thoracic lesions. Other spine and limb injuries were common with cervical, thoracic, and lumbar lesions.

This study included 89 patients (67 men, 22 women) with a mean age of 62.4 ± 1.5 years (Fig. 1). Among them, 58 (65.1%) presented with 223 multiple injuries. The monotrauma and polytrauma groups comprised 74 and 15 patients, respectively. Five (33.3%) patients with fractures requiring open reduction or internal fixation were included in the polytrauma group. Thoracic injuries were common in the aforementioned patients. Other spinal and limb injuries were also common in patients with thoracic and lumbar injuries (Fig. 2A–C).

The characteristics of SCI patients with monotrauma and polytrauma are displayed in Table 1. No significant differences in the injury causes were observed. The proportion of cervical lesions was significantly higher in the monotrauma versus polytrauma group (*P* = .003). The prevalence of thoracic lesions was significantly higher in the polytrauma versus monotrauma group (*P* = .006). Conversely, the mean SCIWORA score was significantly higher in the monotrauma versus polytrauma group (*P* = .03). No significant intergroup difference was observed in the number of patients who underwent spinal surgery.

**Table 1:** Characteristics of patients with SCI: Monotrauma and Polytrauma.

	Monotrauma (n = 74)	Polytrauma (n = 15)	*P* value
Age, yr	61.8 ± 1.6	58.3 ± 4.3	.122
Sex, male/female, n (%)	58 (78.3)/16 (21.6)	9 (60.0)/6 (40.0)	.187
BMI, kg/m²	22.3 ± 0.3	24.0 ± 1.5	.064
Medical history			
OPLL, n (%)	10 (13.5)	0	.202
DISH, n (%)	11 (14.8)	5 (33.3)	.134
Primary injury level			
Cervical, n (%)	47 (63.5)	3 (20.0)	.003*
Thoracic, n (%)	013 (17.5)	8 (53.3)	.006*
Lumbar, n (%)	014 (18.9)	4 (26.6)	.493
ASIA impairment scale at admission			
A	5 (6.7)	3 (20.0)	.1
B	11 (14.8)	0	.1
C	16 (21.6)	2 (13.3)	.7
D	12 (16.2)	2 (13.3)	1.0
E	30 (40.5)	8 (53.3)	.4
ASIA impairment scale at last follow-up period			
A	30 (40.5)	8 (53.3)	.4
B	12 (16.2)	2 (13.3)	.1
C	16 (21.6)	2 (13.3)	.5
D	11 (14.8)	0	.4
E	5 (6.7)	3 (20.0)	.5
Cause of injury			
Traffic accident, n (%)	013 (17.5)	4 (26.6)	.474
Fall from on high, n (%)	34 (45.9)	9 (60.0)	.4
Fall, n (%)	017 (22.9)	00	.065
Struck by object, n (%)	10 (13.5)	2 (13.3)	1.0
SCIWORA, n (%)	28 (37.8)	1 (6.6)	.03*
ISS, Mean ± SD	16.0 ± 1.0	25.0 ± 2.5	<.001*
Surgical treatment			
For spine, n (%)	39 (52.7)	12 (80.0)	.084
For polytrauma, n (%)	–	5 (33.3)	–
Transferred to rehabilitation hospital, n (%)	62 (83.7)	13 (86.6)	1.0
Total length of hospitalization, days	77.0 ± 26.9	106.0 ± 78.0	.166
Total rehabilitation term, days	104.0 ± 32.6	169.0 ± 80.5	.174
Observation period, days	735.0 ± 98.8	751.0 ± 205.4	.76
Clinical outcomes			
ASIA motor score			
At admission	74.7 ± 4.0	89.0 ± 5.1	.6
At last follow-up period	91.5 ± 2.1	94.1 ± 1.9	.9
The Barthel Index			
At admission	5.0 ± 1.6	0 ± 3.0	.326
At last follow-up period	100.0 ± 4.0	100.0 ± 5.8	.06
EQ5d	0.7 ± 0.01	0.8 ± 0.03	
Return to home living, n (%)	61 (82.4)	13 (86.6)	1.0
Within 90 days	51 (83.6)	5 (38.4)	
Over 90 days	23 (37.7)	8 (61.5)	
Return to work, n (%)	20 (46.5)	5 (71.4)	.3

Values are presented as the mean ± standard deviation (SD).

ASIA = American Spinal Injury Association, BMI = body mass index, DISH = diffuse idiopathic skeletal hyperostosis, EQ5d = European Quality of Life Scale, ISS = injury severity score, OPLL = ossification of the longitudinal ligament, SCIWORA = spinal cord injury without radiographic abnormality.

* *P* < .05, chi-squared test.

The ISS was significantly higher in the polytrauma versus monotrauma group (monotrauma, 16.0 ± 1.0; polytrauma, 25.0 ± 2.5; *P* < .001). No significant intergroup differences were noted in the observation period, total length of hospitalization, or total rehabilitation period. Moreover, the mean hospital stay for acute treatment was significantly longer for the polytrauma versus monotrauma group. (monotrauma, 26.3 ± 1.7 days; polytrauma, 53.1 ± 14.6 days; *P* = .04).

Overall, 74 patients (83.1%) lived at home, 11 (12.3%) were in a care facility, and 4 (4.4%) were in a long-term medical care ward at the last follow-up. Within 90 days, 56 patients (62.9%) returned home, while 18 (20.2%) were in a long-term medical care ward. No significant intergroup differences were observed in the proportion of patients who returned home at the final follow-up (Table 1).

Overall, 50 patients (56.1%) were employed at admission, of whom 25 (50.0%) returned to work by the final follow-up. No significant intergroup differences in the proportions of patients who returned to work at the final follow-up were observed (Table 1).

### 3.2. Association between trauma severity and clinical outcomes

**Figure 3.**
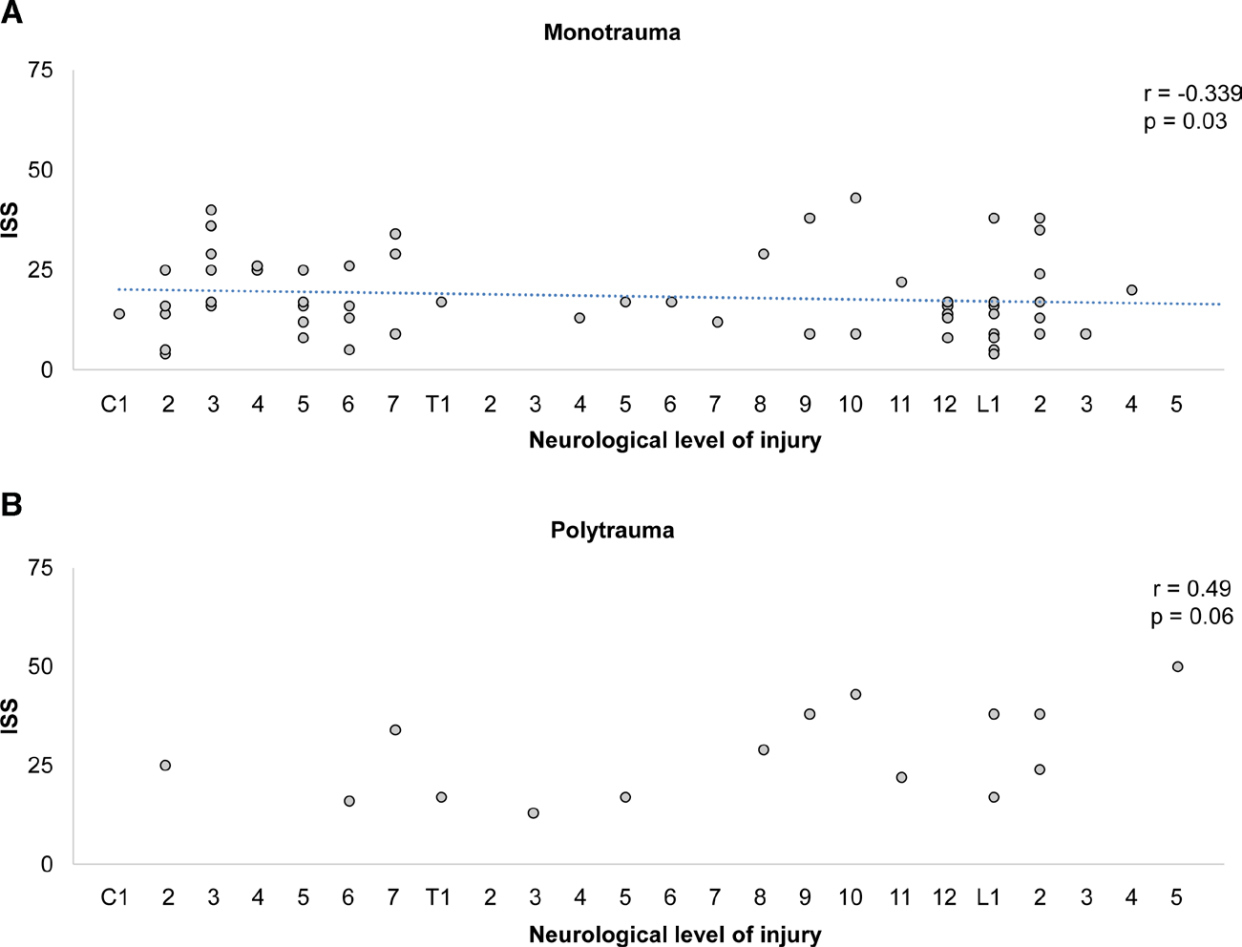
Association between spinal injury level and Injury Severity Score (ISS). There was a significant negative correlation between trauma severity and spinal injury level in the monotrauma group. ISS = Injury Severity Score.

A significant negative correlation between ISS and spinal injury level in the monotrauma group (crude, *P* = .002, *r* = −0.35; adjusted, *P* = .004, *r* = −0.34; Fig. 3A) was present as opposed to the polytrauma group (Fig. 3B). Furthermore, significant negative correlations existed between the ISS and ASIA motor scores at the first visit (crude, *P* < .001, *r* = −0.37; adjusted, *P* < .001, *r* = −0.37) and the last follow-up (crude, *P* = .004, *r* = −0.32; adjusted, *P* = .007, *r* = −0.30). The Barthel Index was also negatively correlated with ISS at the first visit (crude, *P* = .03, *r* = −0.22; adjusted, *P* = .04, *r* = −0.21) and at the last follow-up (crude, *P* = .002, *r* = −0.32; adjusted, *P* < .001, *r* = −0.35). Additionally, ISS was significantly negatively correlated with EQ5d at the last follow-up (crude, *P* = .02, *r* = −0.26; adjusted, *P* = .01, *r* = −0.28; Table 2). The chi-squared test demonstrated that patients with an ISS < 14 returned to living at home (*P* = .03), while patients with an ISS < 19 returned to work (*P* = .02; Table 3).

**Table 2:** Summary of correlation between ISS and outcome.

		At admission		At last follow-up period	
		*P* value	*r*	*P* value	*r*
ASIA motor score	Crude	<.001*	−0.37	.004*	−0.32
	Adjusted	<.001*	−0.37	.007*	−0.30
The Barthel Index	Crude	.03*	−0.22	.002*	−0.32
	Adjusted	.04*	−0.21	<.001*	−0.35
EQ5d	Crude			.02*	−0.26
	Adjusted			.01*	−0.28

ASIA = American Spinal Injury Association, EQ5d = European Quality of Life Scale, ISS = injury severity score.

* *P* < .05, Spearman’s correlation coefficient.

**Table 3:** ISS and return to home living or work.

		ISS		
		<14	14–19	>19
Return to home living	Yes	26 (29.2)	24 (26.9)	24 (26.9)
	No	1 (1.1)	7 (7.8)	7 (7.8)
Return to work	Yes	11 (22.0)	10 (20.0)	4 (8.0)
	No	7 (14.0)	5 (10.0)	13 (26.0)

ISS = injury severity score.

## 4. Discussion

This is the first study to investigate the association between ISS and clinical outcomes in older Japanese patients with SCI. Polytrauma accounted for 16.8% of cases. The mean ISS was significantly higher in the polytrauma versus monotrauma group. Barthel Index and ASIA motor scores at admission and the last follow-up as well as EQ5d at the last follow-up were correlated with ISS. Patients with an ISS < 14 returned to living at home, while those with an ISS < 19 returned to work.

Of the patients included in this study, 58 (65.1%) were diagnosed with SCI and multiple injuries and 15 (18.0%) met the criteria for polytrauma. The incidence of SCI with multiple injuries in Italy and Germany ranges from 55% to 71%.^[2,3]^ The most common causes of traumatic SCI in Japan differ from those reported in other countries. In Italy and Germany, 51.4% to 74% of cases were due to traffic accidents,^[2,3]^ whereas 46.5% of cases in Japan and 48.3% of cases in this study were due to falls from a height.^[1]^ Therefore, the polytrauma rate was low in this study and the SCIWORA rate was considered high (32.6%); moreover, the definition of “polytrauma” was not unified in previous studies.^[14]^ The prevalence of chest and lung injuries with thoracic lesions in SCI patients with multiple injuries was higher than that in patients with cervical and lumbar lesions in Italy.^[2]^ In this study, thoracic and other spinal and limb injuries were common.

In this study, the mean ISS of patients with SCI in the monotrauma and polytrauma groups was 16.0 ± 1.0 and 25.0 ± 2.5, respectively. A previous study in Japan reported an ISS of 15 in 65.3% of patients with traumatic spine injuries in 2004 to 2013.^[15]^ Possible explanations for this discrepancy may be the different causes of injury (in this study, low-energy injury; in previous studies, traffic accidents).

This study clarified that ISS is associated with paralysis, activities of daily living, quality of life, and lifestyle in the aging Japanese society. In previous studies, ISS was linked to the ASIA motor score,^[6]^ functional independence measure,^[6]^ EQ5d,^[16]^ return to living at home, and^[7]^ return to work.^[6]^ One possible explanation for the aforementioned associations is that ISS is related to SCI paralysis severity. In this study, 57.0% of the ISS were attributed to spinal trauma. Therefore, paralysis severity was strongly associated with ISS in the monotrauma and polytrauma groups. Regarding SCI paralysis severity, a significant correlation was observed between ASIA motor score and ISS.

This study had several limitations. First, the sample consisted of patients at a single emergency and critical care center in Japan and was subject to biased patient selection. The sociodemographic characteristics in this study differed from those previously reported in Japan by Miyakoshi et al^[1]^ Our study population was in an area subjected to heavy snowfall geographically located in a predominantly farming village district, and 22 (24.7%) patients were injured in agricultural work involving growing apples or shoveling snow. Therefore, our results may not be representative of the general Japanese population. Second, the sample size was small, and we could not separately analyze each lesion level or ASIA Impairment Scale grade, as in a previous study.^[2]^ All 38 patients with ASIA Impairment Scale grade E had spinal fractures; of them, 30 had other injuries. To investigate the ISS distribution, we did not exclude ASIA Impairment Scale grade E. In addition, in a previous study, the proportion of ASIA Impairment Scale grade E in Japan was reportedly 35%^[17]^; therefore, understanding their outcomes is essential. However, patients with mild limb numbness may not have visited a hospital, which may have affected the results of the analysis. Finally, non-clinical factors, including universal public health and long-term care insurance systems, and welfare services for individuals with disabilities in Japan. This has enabled renovations for those returning home, allowing patients with SCI to receive long-term rehabilitation. Additionally, patients with SCI underwent rehabilitation for a mean of 218.4 ± 36.5 days. Whether the patients were categorized or not, SCI patients with a Barthel Index oer 67.5 points were provided rehabilitation.

In conclusion, this study investigated the clinical outcomes of traumatic SCI using 10-year data from 2010, the background of which reflects the aging Japanese society. The results suggest that the outcomes of the aging society of Japan can be predicted using ISS evaluations at the time of traumatic SCI.

## Acknowledgments

The authors thank Drs Hitoshi Kudo and Sunao Tanaka of the Department of Orthopedic Surgery, Hirosaki University Graduate School of Medicine for their technical support and valuable discussions. We would like to thank Editage (www.editage.com) for the English language editing.

## Author contributions

**Conceptualization:** Manami Tsukuda, Gentaro Kumagai, Eiichi Tsuda.

**Data curation:** Manami Tsukuda, Gentaro Kumagai.

**Formal analysis:** Manami Tsukuda, Gentaro Kumagai, Eiichi Tsuda.

**Funding acquisition:** Yasuyuki Ishibashi.

**Investigation:** Manami Tsukuda, Gentaro Kumagai, Kanichiro Wada, Toru Asari, Yohshiro Nitobe, Eiichi Tsuda.

**Methodology:** Manami Tsukuda, Gentaro Kumagai.

**Project administration:** Gentaro Kumagai.

**Supervision:** Yasuyuki Ishibashi.

**Visualization:** Gentaro Kumagai.

**Writing – original draft:** Manami Tsukuda, Gentaro Kumagai.

**Writing – review & editing:** Manami Tsukuda, Gentaro Kumagai, Eiichi Tsuda, Yasuyuki Ishibashi.
